# Mineral and Matrix Changes in Brtl/**+** Teeth Provide Insights into Mineralization Mechanisms

**DOI:** 10.1155/2013/295812

**Published:** 2013-05-29

**Authors:** Adele L. Boskey, Kostas Verdelis, Lyudmila Spevak, Lyudmila Lukashova, Elia Beniash, Xu Yang, Wayne A. Cabral, Joan C. Marini

**Affiliations:** ^1^Musculoskeletal Integrity Program, Hospital for Special Surgery, 535 E 70th Street, New York, NY 10021, USA; ^2^Department of Endodontics, School of Dental Medicine, University of Pittsburgh, 3501 Terrace Street, Pittsburgh, PA 15261, USA; ^3^Department of Oral Biology, School of Dental Medicine, University of Pittsburgh, 3501 Terrace Street, Pittsburgh, PA 15261, USA; ^4^Bone & Extracellular Matrix Branch, NIH/ NICHD, Bethesda, MD 20892, USA

## Abstract

The Brtl/+ mouse is a knock-in model for osteogenesis imperfecta type IV in which a Gly349Cys substitution was introduced into one COL1A1 allele. To gain insight into the changes in dentin structure and mineral composition in these transgenic mice, the objective of this study was to use microcomputed tomography (micro-CT), scanning electron microscopy (SEM), and Fourier transform infrared imaging (FTIRI) to analyze these structures at 2 and 6 months of age. Results, consistent with the dental phenotype in humans with type IV OI, showed decreased molar volume and reduced mineralized tissue volume in the teeth without changes in enamel properties. Increased acid phosphate content was noted at 2 and 6 months by FTIRI, and a trend towards altered collagen structure was noted at 2 but not 6 months in the Brtl/+ teeth. The increase in acid phosphate content suggests a delay in the mineralization process, most likely associated with the defect in the collagen structure. It appears that in the Brtl/+ teeth slow maturation of the mineralized structures allows correction of altered mineral content and acid phosphate distribution.

## 1. Introduction 

Osteogenesis imperfecta (OI) and dentinogenesis imperfecta (DGI) are rare genetic diseases associated, for the most part, with abnormalities in collagen structure, production, or processing, which result in fragile (brittle) mineralized tissues [1–4]. In addition to the four types of OI originally described by Sillence et al. [5], additional types have been identified in humans [6], and their underlying molecular origins for the most part have been determined. The reasons for the variation in mineralization and mechanical properties in OI and DGI are less certain. The Brtl/+ mouse is a knock-in model for moderately severe OI in which a Gly349Cys substitution identified in a child with type IV OI was introduced into one COL1A1 allele, resulting in a phenotype representative of type IV OI [7]. Changes in the whole bone mechanical strength and collagen D-spacing in these Brtl/+ mice have previously been reported [8, 9] along with variations in their osteoblast and osteoclast activities [10]. The animals were also noted, based on light microscopy, to have a DGI phenotype at 2 months due to changes in the pulp cavities of their teeth [7]. The purpose of the present study was to gain further insight into the mineralization and collagen defects in Brtl/+ mouse teeth using a combination of Fourier transform infrared microscopic imaging (FTIRI), scanning electron microscopy (SEM), and microcomputed tomography (micro-CT) and to use this information to elucidate the mechanism underlying these defects.

## 2. Materials and Methods

### 2.1. Animals

The Brtl/+ mice were derived as described [7] and sacrificed for other purposes under an NIH-approved IACUC approval at two and six months. The mice were fed a standard (NIH 31) chow and were given sterilized fluoridated tap water, ad libitum. Brtl/+ mice have a mixed background of Sv129/CD-1/C57BL/6S and are bred by crossing heterozygous Brtl/+ with WT [7]. Based on preliminary analyses using GraphPad StatMate 2.0 (GraphPad Corp, San Diego, CA, USA) it was determined that to detect a significant difference with a power of 80% in mineral/matrix content by FTIRI, six teeth would be needed at 2 months of age. Jaws (*n* = 6 per genotype), provided frozen, were fixed in ethanol for micro-CT analyses. Jaws were then embedded in polymethyl methacrylate (PMMA) for backscattered SEM and Fourier transform infrared imaging (FTIRI).

### 2.2. Micro-CT

Three-dimensional architecture and geometry of both the crowns and roots of first and second molars were determined by microcomputed tomography using a Scanco *μ*CT35 (Scanco Medical, Basselrsdorf, Switzerland) system with a 12 *μ*m voxel size at 55 kVp as detailed elsewhere [11, 12]. Crown and root regions of interest were generated by defining the respective contours on sequential reconstructed volume slices [12, 13]. Because of clear and consistent respective interfaces, the segmentation of dentin from the background and enamel from dentin was based on 0.6 and 1.7 g/cc, respectively, global thresholds. The parameters evaluated were tissue volume (TV-representing the total crown or root volume including the pulp or root canal space), enamel + dentin volume (BV), the volumes of Enamel and Dentin separately (EV and DV), the mineral density for each fraction (TMD (E) and TMD (D)), and the tissue volume fraction (BV/TV). All parameters were evaluated on the molar 3D volumes using the crown and root regions of interest through the Scanco evaluation software operating in an open VMS environment. Mineral density for dentin was calculated on basis of the dentin/background segmentation value as a minimum threshold and the dentin/enamel one as a maximum threshold, while the enamel mineral density was calculated using only the dentin/enamel segmentation value as a minimum threshold with maximum threshold kept as the highest available in the scale. The Scanco system uses built-in beam energy-specific algorithms for the conversion of attenuation coefficient into mg/cm^3^ values. 

### 2.3. Backscattered Scanning Electron Microscopy (BS SEM)

First and second mandibular molars were embedded in PMMA resin and polished on a MiniMet polisher (Buehler, Lake Bluff, IL, USA) down to 0.25 *μ*m using series of MetaDi diamond suspensions, as previously described [14]. Three WT and 3 Brtl/+ samples were studied in JEOL 6335F Field Emission SEM equipped with a backscattered electron detector. The microscopy was conducted at 10 KV and at a working distance of 14 mm.

### 2.4. Fourier Transform Infrared Imaging

Multiple sections (1-2 microns) of nondecalcified molars, embedded in PMMA, were examined by Fourier transform infrared spectroscopic imaging (Perkin Elmer model Spotlight 100 imaging system) as detailed elsewhere [15, 16]. In brief, dentin spectral images from the whole crown (sectioned along the sagittal plane) or root areas (the mesial or distal root, imaged separately) from first and second molars were examined at 4 *μ*m spatial resolution. ISYS software (Spectral Dimensions, Olney, MD, USA) was used to process the data, including a subtraction of water vapor and PMMA. Parameters calculated and exhibited as images were (i) mineral-to-matrix ratios (Min/Mat, a comparison of the relative ratios of the integrated intensities of the v1, v3 phosphate band, ~900–1200 cm^−1^ to that of the protein amide I band (centered at 1660 cm^−1^)), (ii) carbonate (855–890 cm^−1^) to phosphate band area ratio (CO_3_/PO_4_, indicating carbonate substitution for phosphate in the mineral) (iii) crystallinity (XST, an estimate of crystallite size and perfection, based on the proportion of stoichiometric and nonstoichiometric apatite), (iv) collagen maturity (XLR, a peak height ratio of subbands in the collagen amide I peak), and (v) acid phosphate content HPO_4_, (1128 cm^−1^/1096 cm^−1^) an estimate of the amount of acid phosphate substitution in the mineral lattice. The validation of these parameters is described in detail elsewhere [15–17].

### 2.5. Statistics

The mean values and standard deviations for each tissue type and each parameter in each animal were calculated and the values were compared by ANOVA between Brtl/+ and WT crown and root areas, followed by a Bonferrni multiple comparison test. Due to the limited number of animals available for analysis no correction for animal sex was made. Where multiple comparisons of data were involved, a *P* < (0.05/*n*), where *n* is the number of comparisons, was considered significant.

## 3. Results

**Figure 1:**
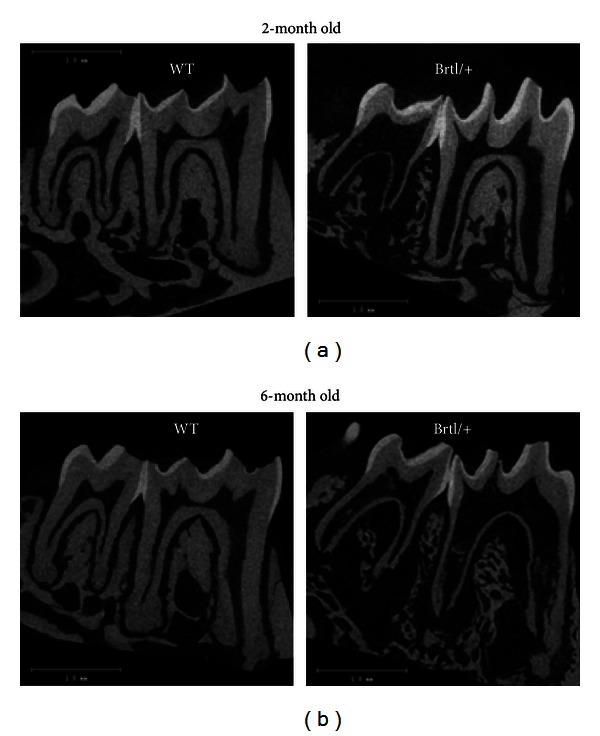
Sagittal views from micro-CT images of Brtl/+ and WT first and second molars at (a) 2 and (b) 6 months. Brtl/+ images show wide pulp spaces and decreased dentin thickness, more pronounced in the root, compared to age matched WT. Note the disorganized trabecular jaw bone around the Brtl/+ roots.

The molars of the Brtl/+ animals differed from those of the WT when examined by micro-CT (Figures 1(a) and 1(b)) with small but significant (*P* < 0.01) decrease in the first and second molar Brtl/+ crowns and root total volume and dentin volumes relative to WT at 2 months and 6 months (Table 1). This decrease of dentin thickness was most pronounced in the root. The mineralized tissue volume fraction (BV/TV) was decreased in the root of the Brtl/+ molars at both 2 and 6 months. There was a small, not consistent increase in the first and second molars, crowns and root mineral density in the Brtl/+ molars at 2 months. As previously noted [7], both first and second molars had widened pulp spaces and a disorganized bone structure around the molar roots.

**Figure 2:**
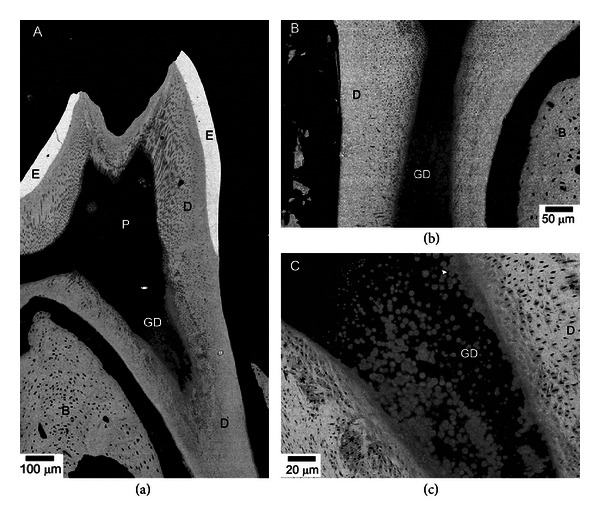
BS SEM micrographs of Brtl/+ mandibular molars. (a) Low resolution micrograph showing overall morphology of the tooth. Note an area of globular dentin (GD) in the cervical root. (b) A micrograph of a molar from a different animal also featuring GD. (c) Closeup of the area containing GD from (a). Note the variations in the degree of mineralization. B: bone, D: dentin, E: enamel, and P: pulp. Asterisks mark undermineralized areas.

**Figure 3:**
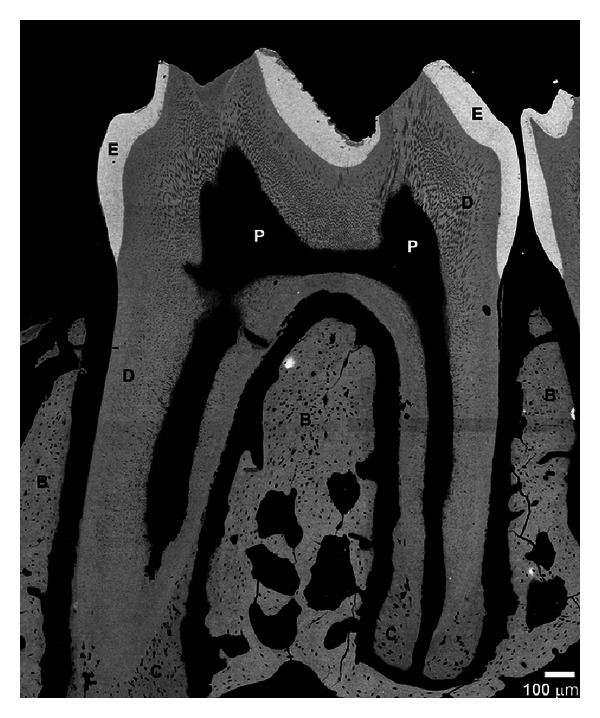
BS SEM micrograph of WT mandibular molars. Abbreviations are as in Figure 2.

No major morphological difference between Brtl/+ (Figure 2) and wild type (Figure 3) mandibular molars was observed by backscattered electron imaging, based on the analysis of 3 samples per group. The density and organization of dentinal tubules were similar in WT and Brtl/+ molars. The reduced dentin thickness and increased pulp chamber size in Brtl/+ molars observed in the micro-CT 3-dimensional volumes were not noticeable in the SEM images. Only one tooth had an abnormal mesial root with thin root dentin and a wide root canal. Interestingly, the presence of globular dentin was observed in the cervical portions of the roots of all Brtl/+ molars (Figure 2), while no globular dentin was detected in wild type molars (Figure 3). At higher magnifications, however, significant abnormalities in dentin mineralization in Brtl/+ were observed. Specifically, noticeable variations in the dentin density were observed in the ~50 *μ*m thick proximal dentin layer adjacent to the pulp cavity (Figure 2(c)). Additional studies are needed to further investigate this phenomenon.

**Figure 4:**
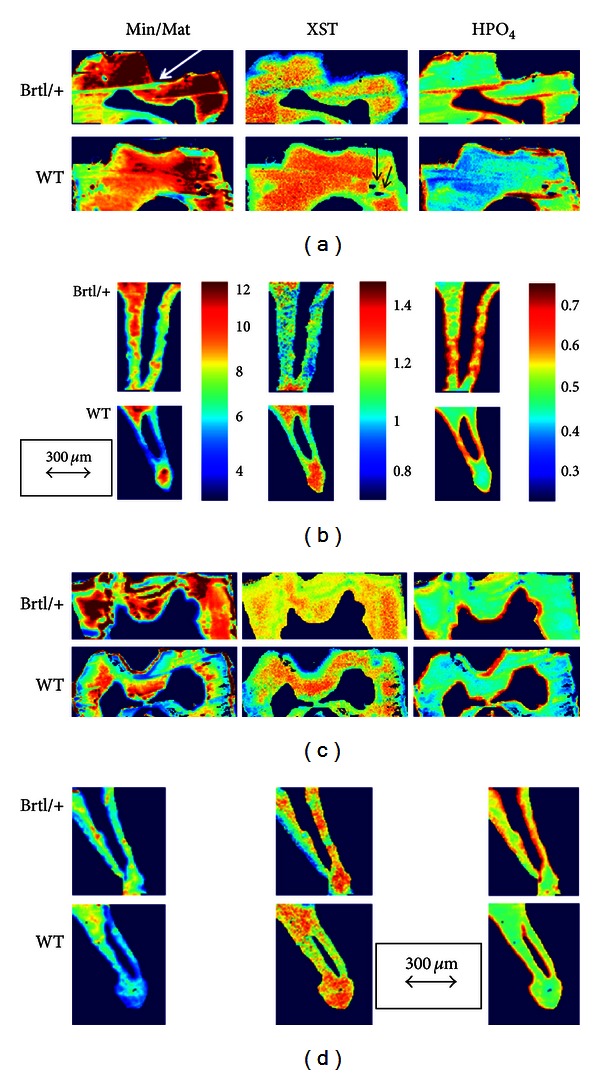
Typical FTIRI images for Brtl/+ and WT molars showing mineral/matrix ratio (Min/Mat), crystal size and perfection (XST), and acid phosphate content (HPO_4_) in (a) first molar crowns at 2 months; (b) second molar root at 2 months; (c) first molar crowns at 6 months; and (d) second molar root at 6 months. The arrows indicate the distance corresponding to 300 *μ*m. The color bars shown for (b) are the same for all similar parameters in the figure. White arrow indicates folds in specimen while black arrows show holes in the replicate images. These areas were masked when calculating numeric data.

Fourier transform infrared imaging (FTIRI) of the molars showed the average mineral/matrix ratio not being significantly altered in the Brtl/+ molars at both 2 and 6 months (Table 2). In fact, the only significant differences in the mean values of the FTIRI images of the teeth were in the acid phosphate content, which was increased in the Brtl/+ roots and crowns at both ages. The distribution of mineral/matrix ratio, crystallinity, and acid phosphate content looked different in typical images of the molars and roots (Figure 4). These “chemical photographs” [18] show, as is typical for mouse molar sections, a broad distribution of mineral properties with visibly increased mineral/matrix ratio in the Brtl/+ molars, as shown in terms of standard deviation in Table 2. The sharp lines and small holes in the section (indicated by arrows) are an artifact due to folds and actual holes made during sectioning and were not included when the average values were calculated. The increased acid phosphate accumulation was apparent in the images (far right column, Figure 4). In the 2-month-old animals the mean crystallinity tended to be reduced in the Brtl/+ crowns and roots, and at 6 months there were some differences in CO_3_/PO_4_ ratio, but the most significant differences were in the acid phosphate substitution.

## 4. Discussion

Dentin and enamel mineralizations occur on different types of matrices. The enamel matrix mineralization is influenced by globular proteins such as amelogenin and enamelin [19, 20] which constitute a small fraction of the final mineralized matrix. Dentin mineralization, by contrast, is controlled by the fibrous protein collagen and its associated noncollagenous proteins [21] which persist throughout the lifetime of the tissue. Many of the noncollagenous proteins are intrinsically disordered and assume conformations to match their binding partners [22], such as collagen and hydroxyapatite mineral. In the Brtl/+ molars, the defect in the collagen structure [9] may affect the binding and the concentration of these noncollagenous proteins, their synthesis by collagen producing cells, and their ability to regulate mineralization, as occurs in both Brtl/+ stem cells and bone [23]. This is seen in the data at both 2 and 6 months, where the teeth are smaller and the mineral properties of the dentin, but not the enamel, are distinct from those of the wild type.

There is only one report of changes in mouse molar micro-CT parameters with age, in which fibromodulin-deficient mice at 3 weeks and 10 weeks were compared to wild type mice, and a reversal of phenotypic differences was found [24]. It is not clear from only two time points in each of the fibromodulin studies whether older animals begin to show degradation of their teeth or whether the KO animal's molars simply begin to approach the WT values as they age. This is one of the limitations of our Brtl/+ study as we only studied animals at 2 and 6 months. It might have been interesting if we had included teeth from Brtl/+ mice at a younger age. The increases in mineralization of teeth with age, along with the altered collagen structure, imply that the hypermineralization in this variant of classical OI is associated with an abnormal collagen matrix and provide one possible mechanism for the way these abnormal collagen matrices could affect initial mineralization. In the mice studied here, the 2-month-old Brtl/+ mice have completed their tooth development and have been weaned for about a month. Thus they are chewing their food, and the phenotypic difference between their less mineralized teeth and those of the wild type animals may be more apparent than those which are noted at 6 months when age-related physiologic effects (grinding of the teeth, change in curvature due to alveolar osteoclasts, etc. [25]) may mask the Brtl/+ phenotype.

Our data represent the first quantitative evaluations of the mineral properties of the teeth in Brtl/+ mice. Earlier studies had reported the presence of widened and infected pulp cavities characteristic of dentinogenesis imperfecta in patients with osteogenesis imperfecta [7]. Although age-dependent changes were noted in both WT and Brtl/+ molars, the absence of studies monitoring age-dependent change in healthy mice teeth during development made it difficult to comment on the effect of the transgene in this study. However there is definitely a suggestion of impaired matrix formation which in turn leads to the persistence of a more acid phosphate containing mineral phase in the Brtl/+ molars at both ages. Acid phosphate is present to a greater extent in newly deposited mineral [16]. The observation that no significant change in mineral/matrix ratio was detected in the FTIRI analysis of the molars agreed with the finding of no change in dentin or enamel mineral density but may indicate that these tissues were already fully mineralized by 2 months as suggested by a comparison of the mineral/matrix ratios of comparable sites in wild type teeth at 2 and 6 months. The presence of globular dentin in the cervical areas of the roots and density variations in the proximal dentin of Brtl/+ detected by SEM is suggestive of mild dysplasia associated with dentinogenesis imperfecta type I (DGI-I), a dental manifestation of osteogenesis imperfecta [26–28]. Although a globular mineralization pattern can be found in normal teeth, where interglobular areas eventually mineralize, extensive areas of globular dentin are often associated with biomineralization abnormalities such as vitamin D deficient X-linked hypophosphatemia, vitamin D deficiency [29–31], and DGI-I.

Brtl/+ mice demonstrate bone fragility, a moderately deformed skeleton, and a low ductility phenotype, accurately representing the biomechanical phenotype of OI as a disease [8]. Their bones also sustain more microdamage than the WT [32]. The respective failure of the odontoblasts to make the physiologic type I collagen trimer results in dental abnormalities and tooth discolorations in patients with all types of the classic OI types [33]. Since we did not observe any enamel wear, we think that the mouse diet had no effect on the 2-month or 6-month molar phenotype. 

The overall mechanism of biologic hydroxyapatite formation is similar in dentin and bone [21, 34, 35]; however, the dental and bone phenotypes can be expected to differ, to some extent because bone is remodeled, and dentin, in general, is not. Future TEM or AFM studies using a fluid cell to provide a source of ions should examine age dependent changes in Brtl/+ teeth and bones to separate effects of mineral nucleation and mineral propagation [36]. Thus the increased remodeling caused by increased RANKL expression in the Brtl/+ mice bones [10] may accentuate the bone phenotype compared to the dentin, but it may also affect alveolar bone remodeling and similarly affect the dentin. It is important to note that the RANKL/OPG ratio in the Brtl/+ mice is normal. Study of the dentin phenotype indicates an impaired matrix mineralization and a slower correction of the phenotype with age, implying that both the collagen matrix and the noncollagenous proteins that regulate the function of that matrix may be altered in the Brtl/+ teeth.

This study has several limitations. Firstly, the Brtl/+ mice were heterozygous rather than homozygous for the mutant allele, resulting in greater heterogeneity of matrix collagen including forms with no, one, or two mutant *α*1(I) alleles. This heterogeneity may contribute to greater variation in the data for mutant as opposed to wild type mice. Nonetheless, significant differences were seen in some critical parameters that can help explain the phenotype. Secondly, animals were studied at 2 and 6 months; thus, initial developmental time points were missed. However, all the earlier studies on the Brtl/+ mice bones used 2- [8–10, 18] and sometimes both 2- and 6-month-old animals [8, 10], and the FTIRI and micro-CT data for the teeth were limited to jaws from these earlier studies. The use of frozen jaws precluded histologic evaluation beyond that already published [7]. The use of jaws from previous studies also prevented observation of the molars as they erupted during their early development. 

## 5. Conclusions 

In conclusion, this imaging study of Brtl/+ teeth demonstrated decreased molar volume and reduced mineralized tissue volume in the teeth with the mutant collagen. The Brtl/+ molar dentin was also thinner. As expected there were no changes in enamel properties demonstrating the different mechanisms involved in collagen-based dentin mineralization and collagen-free enamel mineralization. Increased acid phosphate content was noted at 2 months by FTIRI, and altered collagen structure was noted at 2 but not 6 months in the Brtl/+ teeth.

## Figures and Tables

**Table 1:** Micro-CT parameters measured in Brtl/+ and wild type molars at 2 and 6 months.

	Two months				Six months			
	First molars		Second molars		First molars		Second molars	
	Brtl/+	WT	Brtl/+	WT	Brtl/+	WT	Brtl/+	WT
Crown								
BV (D + E) mm^3^	0.552 ± 0.0002^a^	0.578 ± 0.0003	0.286 ± 0.0003^a^	0.342 ± 6.71*E* − 05	0.516 ± 0.0015	0.552 ± 0.0004	0.293 ± 1.44*E* − 05^a^	0.332 ± 0.0010
BV (D) mm^3^	0.404 ± 0.0001^a^	0.435 ± 0.0005	0.200 ± 0.0001^a^	0.247 ± 0.0001	0.368 ± 0.0007^a^	0.411 ± 0.0002	0.195 ± 1.98*E* − 05^a^	0.235 ± 0.0007
BV (E) mm^3^	0.148 ± 7.69*E* − 05	0.143 ± 7.58*E* − 05	0.086 ± 5.35*E* − 05^a^	0.095 ± 2.99*E* − 05	0.148 ± 0.0002	0.141 ± 2.64*E* − 05	0.098 ± 3.76*E* − 05	0.098 ± 8.37*E* − 05
TMD (D) mg/cm^3^	1327 ± 336^a^	1266 ± 304	1275 ± 1121	1264.9 ± 271	1337 ± 1080	1306 ± 267	1328 ± 1907	1299 ± 295
TMD (E) mg/cm^3^	2024 ± 627	2005 ± 329	1996 ± 382	1995.6 ± 347	2008 ± 1436	2007 ± 244	2002 ± 1346	2006 ± 96

Root								
TV mm^3^	0.482 ± 0.0005^a^	0.544 ± 0.0004	0.243 ± 0.0001^a^	0.309 ± 0.0002	0.557 ± 0.0014^a^	0.610 ± 0.0005	0.294 ± 0.0008^a^	0.355 ± 0.0004
BV mm^3^	0.362 ± 0.0007^a^	0.460 ± 0.0004	0.111 ± 0.0014^a^	0.253 ± 0.0002	0.430 ± 0.0034^a^	0.530 ± 0.0004	0.211 ± 0.025^a^	0.303 ± 0.0004
BV/TV%	0.752 ± 0.003^a^	0.845 ± 0.0002	0.454 ± 0.021^a^	0.820 ± 0.0001	0.773 ± 0.086^a^	0.870 ± 8.03*E* − 05	0.712 ± 0.018^a^	0.853 ± 0.0003
TMD mg/cm^3^	1146 ± 416	1162 ± 406	1056 ± 2187^a^	1131 ± 704	1177 ± 2709	1227 ± 684	1141 ± 2945	1199.4 ± 644

^a^ *P* < 0.0125 versus WT of same age and same tooth type, *n* = 6/genotype.

**Table 2:** FTIRI analysis of Brtl/+ and WT molars (all values are dimensionless ratios).

	Min/Mat	CO_3_/PO_4_	XLR	XST	HPO_4_
2 months					

First molar crown					
WT	10.6 ± 1.0	0.0052 ± 0.0003	4.06 ± 0.20	1.184 ± 0.05	0.44 ± 0.04
BRTL/+	11.0 ± 2.0	0.0051 ± 0.0007	4.07 ± 0.37	1.13 ± 0.03	0.48 ± 0.03
*P*	NS	NS	NS	0.06	0.07

Second molar crown					
WT	10.3 ± 1.0	0.0050 ± 0.0004	4.1 ± 0.29	1.171 ± 0.06	0.450 ± 0.03
BRTL/+	10.6 ± 3.0	0.0050 ± 0.0004	4.2 ± 0.37	1.133 ± 0.04	0.469 ± 0.04
*P*	NS	NS	NS	NS	NS

First molar mesial root					
WT	7.99 ± 0.94	0.0053 ± 0.0004	3.97 ± 0.19	1.175 ± 0.041	0.46 ± 0.03
BRTL/+	7.28 ± 1.54	0.0047 ± 0.0007	4.21 ± 0.39	1.14 ± 0.025	0.53 ± 0.05
*P*	NS	NS	0.08	NS	0.009

Second molar distal root					
WT	6.78 ± 1.0	0.0048 ± 0.0004	4.38 ± 0.89	1.15 ± 0.076	0.51 ± .04
BRTL/+	6.54 ± 0.75	0.0048 ± 0.0006	4.97 ± 0.85	1.09 ± 0.087	0.61 ± 0.05
*P*	NS	NS	NS	0.10	0.001

6 months					

First molar crown					
WT	10.9 ± 2.2	0.0066 ± 0.0005	4.1 ± 0.3	1.1 ± 0.02	0.45 ± 0.02
Brtl/+	13.0 ± 2.6	0.0064 ± 0.0008	4.4 ± 0.7	1.1 ± 0.01	0.49 ± 0.01
*P*	NS	NS	NS	NS	0.015

Second molar crown					
WT	9.0 ± 0.2	0.0063 ± 0.0006	4.1 ± 0.2	1.1 ± 0.01	0.48 ± 0.04
Brtl/+	9.8 ± 1.5	0.0064 ± 0.0007	4.2 ± 1.1	1.06 ± 0.51	0.51 ± 0.05
*P*	NS	NS	NS	NS	NS

Second molar mesial root					
WT	7.1 ± 0.8	0.0062 ± 0.0003	4.2 ± 0.3	1.08 ± 0.03	0.53 ± 0.02
Brtl/+	6.7 ± 1.5	0.0053 ± 0.0004	4.4 ± 0.4	1.09 ± 0.02	0.61 ± 0.15
*P*	NS	0.0009	NS	NS	NS

Second molar distal root					
WT	7.9 ± 0.5	0.0065 ± 0.0004	4.5 ± 0.3	1.08 ± 0.03	0.49 ± 0.03
Brtl/+	7.1 ± 1.5	0.0065 ± 0.0006	4.6 ± 0.4	1.07 ± 0.03	0.59 ± 0.09
*P*	NS	NS	NS	NS	0.07
